# Metallothionein, Copper and Alpha-Synuclein in Alpha-Synucleinopathies

**DOI:** 10.3389/fnins.2017.00114

**Published:** 2017-04-04

**Authors:** Yuho Okita, Alexandre N. Rcom-H'cheo-Gauthier, Michael Goulding, Roger S. Chung, Peter Faller, Dean L. Pountney

**Affiliations:** ^1^Menzies Health Institute Queensland, Griffith UniversityGold Coast, QLD, Australia; ^2^Department of Biomedical Sciences, Faculty of Medicine and Health Sciences, Macquarie UniversitySydney, NSW, Australia; ^3^Centre National de la Recherche Scientifique, Institut de Chimie UMR 7177, Université de StrasbourgStrasbourg, France; ^4^University of Strasbourg Institute for Advanced StudyStrasbourg, France

**Keywords:** Parkinson's disease, α-synuclein, copper, multiple system atrophy, dementia with lewy bodies, metallothionein

## Abstract

Metallothioneins (MTs) are proteins that function by metal exchange to regulate the bioavailability of metals, such as zinc and copper. Copper functions in the brain to regulate mitochondria, neurotransmitter production, and cell signaling. Inappropriate copper binding can result in loss of protein function and Cu(I)/(II) redox cycling can generate reactive oxygen species. Copper accumulates in the brain with aging and has been shown to bind alpha-synuclein and initiate its aggregation, the primary aetiological factor in Parkinson's disease (PD), and other alpha-synucleinopathies. In PD, total tissue copper is decreased, including neuromelanin-bound copper and there is a reduction in copper transporter CTR-1. Conversely cerebrospinal fluid (CSF) copper is increased. MT-1/2 expression is increased in activated astrocytes in alpha-synucleinopathies, yet expression of the neuronal MT-3 isoform may be reduced. MTs have been implicated in inflammatory states to perform one-way exchange of copper, releasing free zinc and recent studies have found copper bound to alpha-synuclein is transferred to the MT-3 isoform *in vitro* and MT-3 is found bound to pathological alpha-synuclein aggregates in the alpha-synucleinopathy, multiple systems atrophy. Moreover, both MT and alpha-synuclein can be released and taken up by neural cells via specific receptors and so may interact both intra- and extra-cellularly. Here, we critically review the role of MTs in copper dyshomeostasis and alpha-synuclein aggregation, and their potential as biomarkers and therapeutic targets.

## Introduction

There is considerable interest in understanding the role of copper in the etiology of PD. Copper homeostasis is disrupted in the PD brain. Furthermore, *in vitro* studies demonstrate that copper can exacerbate alpha-synuclein aggregation (Carboni and Lingor, 2015; Davies et al., 2016). Recent studies have indicated that MTs, one of the major copper regulatory proteins in mammalian cells, may influence alpha-synuclein aggregation in a copper-dependent manner (Adam et al., 2016). This review provides an overview of recent literature describing the role of copper dyshomeostasis in PD and the potential role of MTs in this process.

Several neurodegenerative diseases, including PD, multiple system atrophy (MSA), and dementia with Lewy bodies (DLB), show abnormal folding and aggregation of the alpha-synuclein protein correlating with death of neuronal cells (Barnham et al., 2004; Barnham and Bush, 2008; Wong and Krainc, 2017). The pathogenesis of these disorders is complex; a result of the interplay between genetic susceptibility and environmental factors (Dauer and Prezedborski, 2003; Chai and Lim, 2013). PD is a chronic disease caused by the progressive degeneration of dopaminergic neurons, affecting both motor and non-motor functions (Lees et al., 2009; Jenner et al., 2013). Degeneration progresses from the brain stem to the *locus coeruleus, dorsal* and *raphe nuclei*, and *ventral tegmental area* to the prefrontal cortex (Wirdefeldt et al., 2011; Jucker and Walker, 2013). Age is the most significant risk factor (Dauer and Prezedborski, 2003), but other factors include head trauma or injury and obesity (Lees et al., 2009; Jenner et al., 2013). There are no diagnostic biomarkers for PD, yet it is estimated that the pre-symptomatic disease can extend over 10 years. PD can arise from autosomal dominant mutations associated with the alpha-synuclein gene, *SNCA*, resulting in A53T, H50Q, E46K, A53E, G51D, and A30P variants (Wirdefeldt et al., 2011; Nussbaum, 2017) and has also been linked to alpha-synuclein overexpression, accumulation and aggregation (Shin et al., 2009; Anandhan et al., 2015; Van der Perren et al., 2015). Recently, a point mutation within the Vps35 subunit of the retromer was linked to PD which results in disrupted trafficking of cathepsin D, a protease important for the degradation of alpha-synuclein (Follett et al., 2014).

DLB is characterized by progressive dementia, hallucinations, delusions and memory loss, rigidity, and postural disturbance (Noe et al., 2004), with both PD and DLB characterized by mostly neuronal alpha-synuclein deposits. A third prominent alpha-synucleinopathy is MSA a condition which shows alpha-synuclein inclusions mostly affecting glial cells (Spillantini and Goedert, 2000). MSA is rapidly progressive and shares most of its symptoms with PD, although degeneration occurs both to the central nervous system and periphery (Jellinger, 2014; Radford et al., 2014).

Current treatments for PD do not cure the underlying condition but alleviate symptoms (Dauer and Prezedborski, 2003; Jankovic, 2012; Jenner et al., 2013). Dopamine replacement therapy via the precursor L-DOPA (L-3,4-dihydroxyphenylalanine) is the most prevalent treatment (Jankovic, 2012). However, patients develop resistance to L-DOPA over time (Connolly and Lang, 2014) and prolonged treatment may eventually accelerate deterioration due to generation of reactive oxygen species (Barnham et al., 2004; Thanvi and Lo, 2004), whereas, pre-treatment with non-steroidal anti-inflammatories was found to be effective in animal PD models (McCarty, 2006; Pinto et al., 2016). The development of mechanism-based therapeutics and neuroprotective strategies, including metal chelation, is a focus of on-going research (Hart et al., 2009).

## Lewy bodies and alpha-synuclein

In PD pathology there is an accumulation of alpha-synuclein in the brain stem, spinal cord, and cortex (Lees et al., 2009), primarily in the form of intra-neuronal aggregates (Lewy bodies, LB) composed mostly of alpha-synuclein. Other aggregate proteins including Parkin, PINK-1, DJ-1, and LRRK-2 gene products relate to pathways such as autophagy/mitophagy processes, oxidative stress mechanisms, and mitochondrial survival (Shin et al., 2009). Alpha-synuclein makes up 1% of total brain soluble proteins in humans (Stefanis, 2012), with putative functions in dopamine production, uptake and storage as well as recycling of neurotransmitter vesicles (Sidhu et al., 2004; Marques and Outeiro, 2012; Stefanis, 2012). It is a small protein (14 kDa) existing as an unfolded monomer (Vekrellis et al., 2004; Cremades et al., 2012), although recent work has revealed that it is also present in oligomeric forms (Yates, 2011). Studies on alpha-synuclein knockout mice implicate its role in neurotransmitter regulation (Yavich et al., 2004). The protein may also act as a calcium channel activator and mediator of calcium entry and may increase cell toxicity from oxidative stress (Dryanovski et al., 2013; Rcom-H'cheo-Gauthier et al., 2016).

Current evidence shows alpha-synuclein oligomerizes and forms aggregates within the neuronal cytoplasm that have a deleterious outcome for the neuron and provide a link to neurotoxicity (Vekrellis et al., 2004; Chen and Feany, 2005; Stefanis, 2012). The inhibition of normal cellular function by alpha-synuclein aggregates leads to increased oxidative stress (Cremades et al., 2012), and overexpression of alpha-synuclein caused protein degradation malfunction phenotype in rats (Kirik et al., 2002; Vekrellis et al., 2004). It is unclear whether LB are neurotoxic or neuroprotective. They may be formed to sequester aberrant proteins that would otherwise be neurotoxic. *Drosophila melanogaster* models of PD have demonstrated that increased inclusion body formation is associated with a reduction in overall toxicity to neuronal cells (Chen and Feany, 2005). Similarly, rat models with inhibition of proteasome function show a rise in aggregates but cessation of neuronal death (Sawada et al., 2004). Although several recent reports indicate that LBs are neurotoxic, contributing to cell death *in vivo* with decreased mitochondrial and proteosomal function which causes increased oxidative stress and cell death (Branco et al., 2010). Alternatively, other studies suggest an inverse correlation between cell viability and the quantity of LB-like alpha-synuclein inclusion bodies (Wan and Chung, 2012). Recently, alpha-synuclein has been observed to propagate between neurons, whereby alpha-synuclein spread mimicked the progression of neurodegeneration observed in PD from the *substantia nigra* toward the frontal cortex (Luk et al., 2012). This propagation of alpha-synuclein resembles prion disease spread, where infectious proteins travel between host cells (Jucker and Walker, 2013; Valdinocci et al., 2017).

## Copper and α-synuclein disease

**Figure 1:**
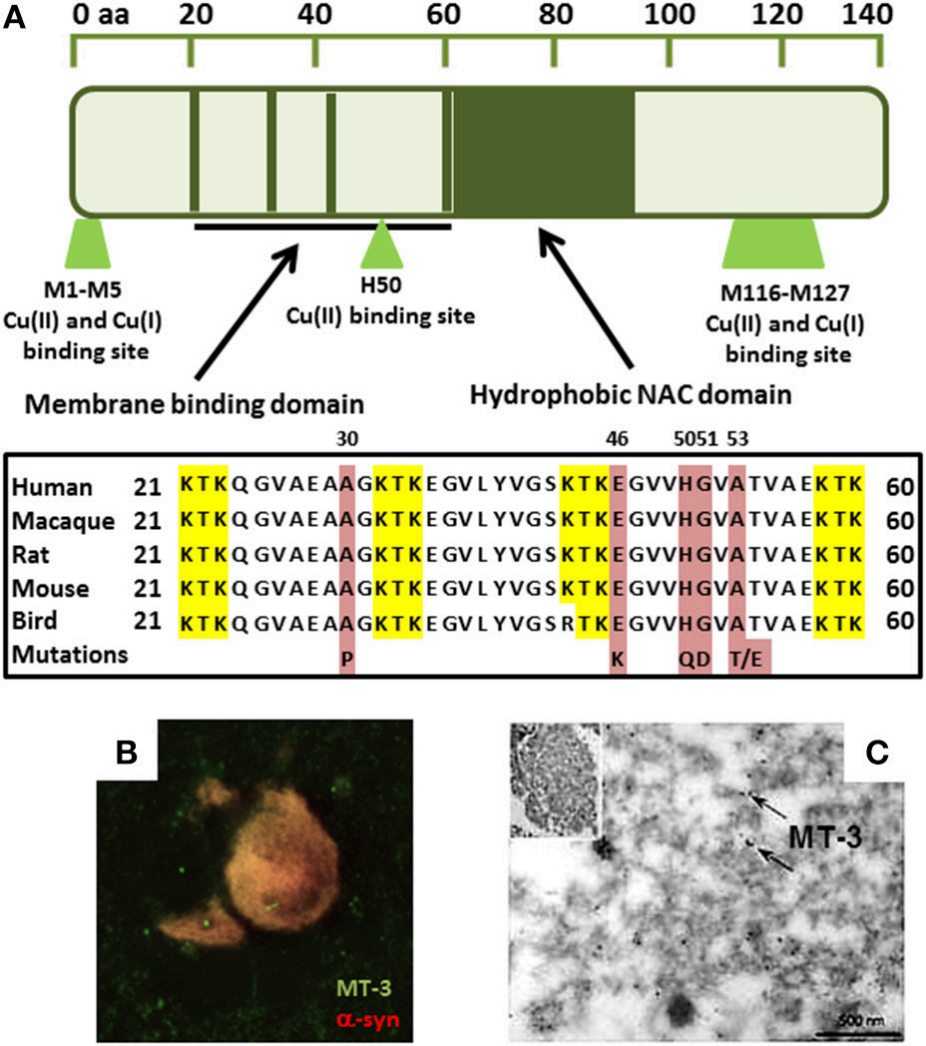
**(A)** Sequence alignment showing alpha-synuclein copper binding ligands overlap with PD-linked H50 mutation in membrane binding domain. Red represents the known point mutations in familial Parkinson's disease in alpha-synuclein. Yellow represents the KTK repeats in the N-terminus which are involved in lipid interaction. **(B)** Immunofluorescence shows MT-3 co-localizes with alpha-synuclein in glial cytoplasmic inclusions in MSA brain tissue. **(C)** MT-3 associates with alpha-synuclein filaments in isolated alpha-synuclein inclusions by immunogold-labeling (black dots; arrows) (Pountney et al., 2011).

Copper is an essential element, mainly as a catalytic center of several enzymes involved in mitochondrial energy transformation, the synthesis of neurotransmitters, cell signaling, and other crucial processes. It accumulates in the brain with aging (Zatta et al., 2008; Vasudevaraju et al., 2010; Pushkar et al., 2013) and has been shown to bind alpha-synuclein and initiate its aggregation (Paik et al., 1999; Rose et al., 2011; Carboni and Lingor, 2015). At physiological pH, two copper(II) binding sites exist on alpha-synuclein corresponding to M1-D2, and H50. However, at pH 5.0, the H50 binding is strongly diminished and alpha-synuclein additionally showed a completely different site for copper binding at D119–E123 (Drew et al., 2008; De Ricco et al., 2015a). Two regions have been shown to bind to copper(I): M1–M5 and M116–M127 (Camponeschi et al., 2013) with M1–M5 being the stronger and thus more relevant (Figure 1). Within the cell, both copper species co-exist and the transition from copper(II) to copper(I) in amyloid aggregates was suggested to contribute to the presence of ROS leading to cell damage (Miotto et al., 2014). The capacity of copper to bind to alpha-synuclein at H50 could also be highly relevant for disease pathology in humans. Indeed, the point mutation H50Q which leads to a familial form of PD corresponds to the major copper(II) binding site (Proukakis et al., 2013) and binding of copper(II) to this mutant results in a structurally different species compared to the copper-WT species (Villar-Piqué et al., 2016). The ability of copper to accept or donate electrons implicates it in the production of ROS in PD (Barnham et al., 2004; Barnham and Bush, 2008; Valensin et al., 2016), and it has more recently been shown that in the SN, total tissue copper is decreased (Montes et al., 2014). Moreover, some studies have found an increase of serum copper, and indeed a positive correlation between high concentration levels and severity of the disease (Brewer, 2009; Arnal et al., 2010). Furthermore, free copper levels, both in PD cases and DLB cases, were found to have increased in the cerebrospinal fluid (CSF) (Pall et al., 1987; Magaki et al., 2007; Hozumi et al., 2011).

Copper in extracellular fluid bound to copper-binding proteins, such as ceruloplasmin, is less harmful than the free, or loosely bound ions (Squitti et al., 2016). These metal-binding proteins act to sequester the reactive metal and most copper present in brain tissue is bound to transport carriers or other proteins (Davies et al., 2013; Squitti et al., 2016). Davies et al. found that neuromelanin-bound copper in the SN of PD patients decreased, suggesting increased free copper linked to neuronal death. It was also found that the copper transporter CTR-1 becomes drastically reduced in the SN of PD brain before regional cell loss begins (Davies et al., 2014). Intracellular copper levels are regulated by the action of membrane copper-ATPases (Cu-ATPases) that pump copper. Genetic mutation of Cu-ATPase (ATP7A) has been found to cause diseases of copper deficiency in the brain and the specific mutation of ATP7B results in diseases of copper accumulation (Lutsenko et al., 2007; Choi and Zheng, 2009; Davies et al., 2016). Recent work has examined copper chelation as a means to reduce free-radical damage, with clioquinol (a metal chelator) shown to reduce aggregation of beta-amyloid and alpha-synuclein in transgenic mice (Cherny et al., 2001; Adlard et al., 2008; Finkelstein et al., 2016). Metallothioneins (MT), contribute to copper regulation and may also act as ROS absorbers (Carter et al., 1984; Otsuka, 2004; Lutsenko et al., 2007). Additionally, MTs have been implicated in MSA (Hozumi, 2013) and the MT-3 isoform was found to co-localize with alpha-synuclein and coat alpha-synuclein filaments in the hallmark glial alpha-synuclein deposits (Figures 1B,C; Pountney et al., 2011). Furthermore, MT-3 was found to inhibit ROS production by copper-alpha-synuclein by a metal-swap mechanism (Meloni and Vašák, 2011).

## Metallothioneins

MTs, are small (6–7 KDa) native Zn/Cd binding proteins (Margoshes and Vallee, 1957; Kägi and Vallee, 1960), composed of cysteine-metal cores that have a high affinity for copper (Pountney et al., 1995; Nordberg, 1998; Hozumi et al., 2004; Carpenè et al., 2007; Dong et al., 2015). MT-knockout mice become extremely susceptible to the effects of environmental stress, including copper-exposure (West et al., 2008; Manso et al., 2011; Petro et al., 2016). MTs occur in two major isoforms: MT-1/2 and MT-3 (Nordberg, 1998), with MT-1/2 present throughout the body, while MT-3 is present primarily in the brain (Palmiter et al., 1992). MT-3 differs by a unique acidic insert comprised of six amino acids and two Pro substitutions and has an observed ability to inhibit cell growth in cultured neurons (Palmiter et al., 1992; Chung et al., 2003; Howells et al., 2010). Recent work showed MT to be neuroprotective against proteinopathies in neurodegenerative disease, a finding which is reinforced by low MT-3 levels in neurons of Alzheimer's patients compared to healthy individuals (Kimura and Itoh, 2008; Howells et al., 2010; Uchida, 2010). Interestingly, this same deficiency is seen in PD, although this finding may be somewhat controversial (Hozumi et al., 2004). Previous work showed that MTs were more highly expressed in Parkinsonian astrocytes (Michael et al., 2011) and also in astrocytes in MSA (Pountney et al., 2011).

MTs bind copper with high affinity, suggesting a role in copper homeostasis (Chung et al., 2008, 2010), and mice displaying copper overload crossed with an MT 1/2 knockout did not survive past infancy (Kelly and Palmiter, 1996). Exchange between protein and metal has been observed in studies that found MTs prevented copper-induced aggregation of amyloid beta peptide or alpha-synuclein (Meloni et al., 2008; Meloni and Vašák, 2011; De Ricco et al., 2015b). The MT gene promoter contains a metal response element (Richards et al., 1984; Sadhu and Gedamu, 1989; West et al., 1990; Pountney et al., 1995), although the MT-3 isoform does not show metal inducibility (Faller, 2010). The cysteine-rich structure and metal-response element of MTs implicate a possible target for attenuating copper-induced aggregation of alpha-synuclein (West et al., 2008; Waldron et al., 2009). Copper-MT may also represent a potential biomarker for Parkinson's or Alzheimer's disease as MT levels increase in response to zinc, copper, or cadmium and physiological stress (Richards et al., 1984). MTs have been found to be recruited to alpha-synuclein aggregates in MSA (Figures 1B,C; Pountney et al., 2011). Other studies have shown that exogenous MT is readily taken up by neuronal cells and identified that neurons take up MT through the megalin receptor (Fitzgerald et al., 2007; Chung et al., 2008). Indeed, alpha-synuclein may also be taken up by neural cells or may interact extracellularly with MT.

## Oxidative stress and neuroinflammation

Oxidative stress results from incomplete reduction of oxygen resulting in the formation of reactive oxygen species (ROS) and/or a decrease in the degradation of these species by endogenous anti-oxidant systems (Halliwell, 2001; Sas et al., 2007). It is unknown whether oxidative stress damage is a prerequisite of PD pathogenesis, or the result of the disease itself (Blesa et al., 2015). Autosomal recessive mutations involving the Parkin gene inhibit its function as a ubiquitin ligase, affecting protein degradation and turnover mechanisms, as well as mitophagy. This may be an important factor as mitochondrial dysfunction leads to oxidative stress (Kitada et al., 1998; Siddiqui et al., 2016). Additionally, PINK1 disruption results in loss-of-function of mitophagic systems. Mutations in DJ-1 similarly disrupt cellular oxidative stress responses and protein degradation systems (Shin et al., 2009). Moreover, MTs were found as effective ROS scavengers in a PD animal model (Ebadi et al., 2005; Ebadi and Sharma, 2006). MTs are capable of self-protection through maintenance of copper and zinc levels and they also directly protect against ROS (Shiraga et al., 1993). Recently, MTs were shown to inhibit the formation of inclusion body-like structures, Charnoly bodies, mitochondrially formed electron-dense membrane stacks found in neurons, leading to a lower observed incidence of alpha-synucleinopathies (Sharma et al., 2013b; Sharma and Ebadi, 2014a,b). The scavenging ability of MTs to combat ROS leads to a lowering of oxidative stress and therefore a potential role in neuroprotection against PD (Ebadi et al., 2005; Ebadi and Sharma, 2006; Hozumi, 2013).

**Figure 2:**
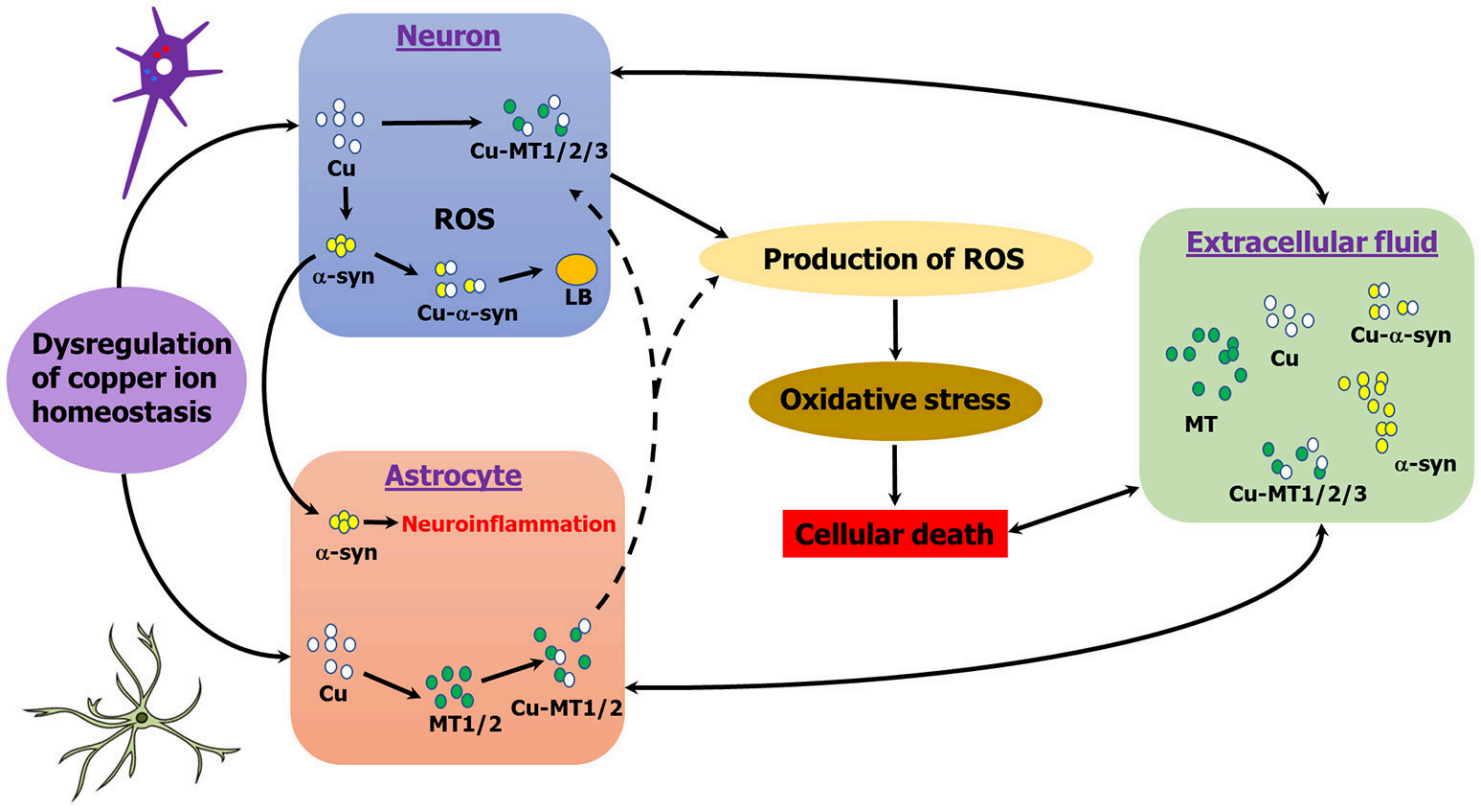
**Schematic representation of copper, MT, α-syn interactions in PD**. MT-1/2 expression is primarily astrocytic in the brain, whereas MT-3 is neuronal. MTs and alpha-synuclein may interact either intra- or extracellularly, either directly or indirectly. Copper induces alpha-synuclein aggregation, whereas MT's chelate copper. Copper binding to alpha-synuclein generates ROS, whereas MTs are powerful anti-oxidants.

Neuroinflammation has been linked to alpha-synucleinopathies and there is evidence of a role of MTs in inflammation in brain injury (Chung et al., 2009; Pedersen et al., 2009; Shastri et al., 2013; Vieira et al., 2015). MTs have been implicated in inflammatory states and act as exchangeable zinc stores which can swap with toxic metals, such as copper and cadmium (Rofe et al., 1992; Meloni et al., 2008). Astrocytes, the primary cell type expressing MT-1/2 in the brain, up-regulate MT-1/2 expression as a physiological response which may promote neuroregeneration as well as survival (Chung et al., 2008; Landowski et al., 2016). Treatment with MT-2 attenuated the immune response of neurotoxic quinolinic acid production in brain injury (Chung et al., 2009). More recently, overexpression of MT-1 in a mouse model of AD promoted reversal in behavioral symptoms of disease and more inert soluble amyloid species (Manso et al., 2016). Indeed, extracellular alpha-synuclein inhibits β-amyloid plaque formation (Bachhuber et al., 2015), a process that may also be influenced by extracellular MT binding (De Ricco et al., 2015b). Further, in a mouse model of AD, MT-3- and MT-1/2-deficient mice had lower plaque burdens and mortality, and performed better in behavioral tests (Manso et al., 2012a,b). Figure 2 summarizes the interplay between MT, alpha-synuclein and copper in different cell types in PD.

## Biomarker and therapeutic potential of metallothionein

The presence of LB post-mortem is the only diagnostic marker for PD (Beach et al., 2013; Sharma et al., 2013a; Espay et al., 2017), although symptoms, such as tremor and rigidity appear after significant damage has already occurred (Jankovic, 2012; Connolly and Lang, 2014). Serum MT levels are significantly increased in pathological conditions including various cancer types (Krizkova et al., 2010) and following traumatic brain injury (Kukacka et al., 2006) and MT can traverse the glomerular apparatus into the urine from the blood supply, with the megalin receptor for MT-1/2 present in the choroid plexus (Lewis et al., 2012). The potential of MT as a biomarker has been speculated in lysosomal storage disorders with nervous system involvement and leukodystrophy (LSDs-LD), where increased white matter MT expression correlated to severity and progression of LSDs-LD and may be related to inflammation and oxidative stress (Cesani et al., 2014). Increased astrocytic MT expression in MSA may similarly point to the biomarker potential of MT in alpha-synuclein disease (Pountney et al., 2011). Indeed, MT is internalized by neural cells via the low-density lipoprotein receptor-related protein receptor, LRP-1, one of the primary trans-membrane transporters involved in the transfer of molecules out of the brain, suggesting that MT might be actively shuttled into the blood (Landowski et al., 2016). Exposure of rodents to heavy metals, such as copper resulted in substantial accumulation of Cu-MT in the liver and kidneys (Kurasaki et al., 1998) suggesting that copper-loaded rather than the natural Cu/Zn-loaded protein in serum or urine could provide a biomarker of copper-imbalance.

Glucocorticoids are regulators of MTs, particularly during fetal development (Quaife et al., 1986) and have been shown to produce increased MT levels in animals (Klaassen, 1981). Dexamethasone is a potent inducer of MT (Karin et al., 1980; Palmiter et al., 1992; Méndez-Armenta et al., 2003), although less effective than heavy metals (Kobayashi et al., 1985), increasing MT-1 mRNA synthesis five-fold (Mayo and Palmiter, 1981) by acting on the glucocorticoid response element in the promoter (Karin et al., 1984; Richards et al., 1984). Dexamethasone, is also neuroprotective via MT induction in an ALS model (Tokuda et al., 2015) and conserved dopamine content by 20% in a PD model (Kurkowska-Jastrzebska et al., 2004). It is tempting to speculate that MT induction could have therapeutic potential in alpha-synuclein diseases.

## Conclusion

MTs may offer neuroprotection in alpha-synucleinopathies, however, further studies are needed to determine if the neuroprotective action is mediated via copper chelation or is associated with ROS scavenging, neuroinflammation or neurotrophic effects, if MT and alpha-synuclein interact directly or indirectly and if this is intra- or extracellular.

## Author contributions

All authors contributed equally to the writing. DP coordinated the manuscript.

### Conflict of interest statement

The authors declare that the research was conducted in the absence of any commercial or financial relationships that could be construed as a potential conflict of interest.

## Abbreviations

AD: Alzheimer's disease
ALP: autophagy lysosomal pathway
ANS: autonomic nervous system
CSF: cerebrospinal fluid
DLB: Dementia with Lewy bodies
MPTP: 1-methyl-4-phenyl-1,2,3,6-tetrahydropyridine (chemical)
MSA: Multiple systems atrophy
MT: metallothionein
PD: Parkinson's disease
ROS: reactive oxygen species
SNpc: substantic nigra, pars compacta
UPS: ubiquitin proteasome system.
